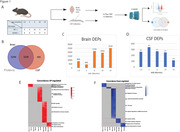# Matched Proteomics of Cerebrospinal Fluid and Brain Reveals Conserved and Discordant Mechanisms of Progressive Aβ Pathology in Mice

**DOI:** 10.1002/alz.091503

**Published:** 2025-01-09

**Authors:** Ali Tfaily, Upasna Srivastava, Sruti Rayaprolu, Prateek Kumar, Hailian Xiao, Duc M Duong, Eric B. Dammer, Nicholas T Seyfried, Srikant Rangaraju

**Affiliations:** ^1^ Yale University School of Medicine, New Haven, CT USA; ^2^ Emory University Center for Neurodegenerative Disease, Atlanta, GA USA

## Abstract

**Background:**

Cerebrospinal fluid (CSF) is an important source of protein biomarkers for diagnosis, risk stratification, and predicting treatment response in Alzheimer's disease (AD). Proximity to brain parenchyma suggests that CSF proteomic alterations may mirror brain pathological changes. Understanding the evolution of CSF proteomic changes and their alignment with concurrent brain pathology necessitates matched CSF and brain analyses, which are possible using animal models of AD pathology.

**Method:**

CSF and brain (cortex) from 86 mice (47 wild‐type (WT) and 39 5xFAD, age groups of 1.8, 3, 6, 10, 14 months, equal males and females) underwent tandem‐mass‐tag mass spectrometry (TMT‐MS) across six TMT batches. After batch‐correction of data, we identified differentially enriched proteins (DEPs, ANOVA p<0.05), comparing WT and 5xFAD across various ages. Gene Set Enrichment Analysis (GSEA) and Gene Set Variation Analysis (GSVA) were conducted to elucidate conserved and discordant pathways in CSF and brain (Figure 1A).

**Result:**

Of 8,535 brain proteins and 3,721 CSF proteins, 3,236 proteins were present in both (Figure 1B). In CSF, most DEPs were found at 1.8‐10‐month ages, followed by a marked decrease at 14 months. In contrast with CSF, DEPs in brain progressively increased with age (Figures. 1C‐1D). GSVA revealed that early unique CSF changes involved synaptic dysfunction and cellular stress, while later changes involved mitochondrial dysfunction and neuronal death. In brain, our analysis indicated heightened immune/glial activation and alterations in cellular structure, with late‐stage changes indicative of cellular, synaptic, and vascular dysfunction. DEPs (5xFAD vs. CSF) in CSF minimally overlapped with brain DEPs (<10%) at 1.8 and 3 months, but increased to >30% at later (6‐14 months) time points. Concordance between CSF and brain changes was highest at 6 and 10 months, particularly in immune response mechanisms and metabolic processes (Figures. 1E‐1F).

**Conclusion:**

Our findings underscore distinct patterns in the CSF and brain proteomes during the progression of Aβ pathology. Although 87% of CSF proteins are shared with brain, limited DEPs overlap shows unique molecular signatures, suggesting that most CSF changes do not reflect brain‐level changes. Despite this, immune, metabolic, and proteostasis‐related mechanisms show temporal concordance between CSF and the brain.